# Identifying the evidence of speech emotional dialects using artificial intelligence: A cross-cultural study

**DOI:** 10.1371/journal.pone.0265199

**Published:** 2022-03-17

**Authors:** Sofia Kanwal, Sohail Asghar, Akhtar Hussain, Adnan Rafique

**Affiliations:** 1 Department of Computer Science, University of Poonch Rawalakot, Rawalakot, Azad Kashmir, Pakistan; 2 Department of Computer Science, Comsats University Islamabad Campus, Islamabad, Pakistan; 3 Department of Computer and Information Science, Higher Colleges of Technology, Abu Dhabi, UAE; UCL: University College London, UNITED KINGDOM

## Abstract

The advancement in technology especially in the field of artificial intelligence has opened up novel and robust ways to reanalyze the many aspects of human emotional behavior. One of such behavioral studies is the cultural impact on the expression and perception of human emotions. In-group advantage makes it easy for the people of the same cultural group to perceive each other’s emotions accurately. The goal of this research is to re-investigate human behavior regarding expression and perception of emotions in speech. The theoretical basis of this research is grounded on the dialect theory of emotions. For the purpose of this study, six datasets of audio speeches have been considered. The participants of these datasets belong to six different cultural areas. A fully automated, machine learning-based framework i.e. Support Vector Machine (SVM) is used to carry out this study. The overall emotion perception for all six cultural groups supports in-group advantage, whereas emotion wise analysis partially supports the In-group advantage.

## 1 Introduction

It is an established finding that non-verbal emotion communication by prosodic means is more precise when expressors and perceivers are from the same cultural group [1]. This is because the cultural differences modulate the expression and perception of emotions [2]. Recent advancements in artificial intelligence have opened up new avenues to study human behavior. Gradual improvement and advancement especially in machine learning have changed the lengthy and expensive research into a precise and less expensive one. Also, there is room for future behavior prediction using Artificial Intelligence (AI) which is not much possible without it [3]. Cross-cultural emotion recognition in voice is an ongoing research area in psychology [4]. However, perceiving regional dialects of human voice by machine learning is more reliable and less expensive method. Automatic emotion dialect identification refers to the identification of the speaker’s regional dialect by machine learning, inside a predefined language, dependent on the prosodic sign and other phonetic range contained inside the speech signal [2]. The AI cut shorts the procedure by avoiding the active involvement of human subjects, lengthy procedures, yet giving the results showing the same behavioral pattern. It uses the already available benchmark datasets so no need to reinvent the wheel. In this sense, the automatic behavior recognition approach is more efficient, effective and comprehensive whereas manual study relied heavily on the involvement of subjects, is laborious, inefficient and subjective [5]. Here in this study, we are deploying a full machine learning-based model to carry out speech emotion recognition and perception task for observing cultural differences. For this purpose, we are using six benchmark datasets representing six different cultures. Each dataset is consisting of audio voices of actors belonging to those regions and speaking the regional dialect. These datasets are: Ryerson Audio-Visual Database of Emotional Speech and Song dataset (RAVDESS) [6], Berlin Emotional Speech Corpus (EmoDB) [7], Sharif Emotional Speech database (ShEMO) [8], Surrey Audio-Visual Expressed Emotion dataset (SAVEE) [9], Italian Emotional Speech Database (EMOVO) [10], and Toronto Emotional Speech Set (TESS) [11]. The machine learning model uses support vector machine for emotion recognition.

In the first step, the preprocessing of each dataset is performed. In the next step, emotion features are extracted using red the openSMILE toolkit. In the third step, the feature reduction technique is applied to reduce the feature dimensionality and ultimately save the classification time. Once the feature sets are prepared, training and testing sets are formulated by seven-fold cross-validation. The recognition task is performed using the SVM model by setting up one language dataset as training and the remaining language datasets as testing. For evaluating the in-group advantage, the Leave-one-subject-out strategy is used within the same culture. Finally, the results are obtained in terms of accuracy and recall. This research has benefits in two ways, i) It introduces a complete machine learning model to explore the dialect theory of emotions. and ii) It gives a deep insight into the emotions of people belonging to different cultural groups and geographies. In order to maintain healthy social interaction in society, it is important to communicate one’s emotional state successfully and conversely identify the exact emotional state of others. Successful social interaction needs perceiving emotions rightly [12]. These findings can be used in the future as a reference to avoid any kind of miscommunication among the population under consideration. The present study is based on dialect theory [13]. It is seen that people belonging to different cultural groups have variation in spoken language dialects. Dialect theory, by using linguistic metaphor, argues that there are subtle differences in expressing and perceiving vocal emotions across cultures. This leads to the observation named: In-group advantage, which means that emotions can precisely be identified by one’s own cultural group as compared to other’s cultural group. The current study was designed to provide the empirical evidence of the theory using machine learning approach.

### 1.1 Theoretical underpinning

**Fig 1:**
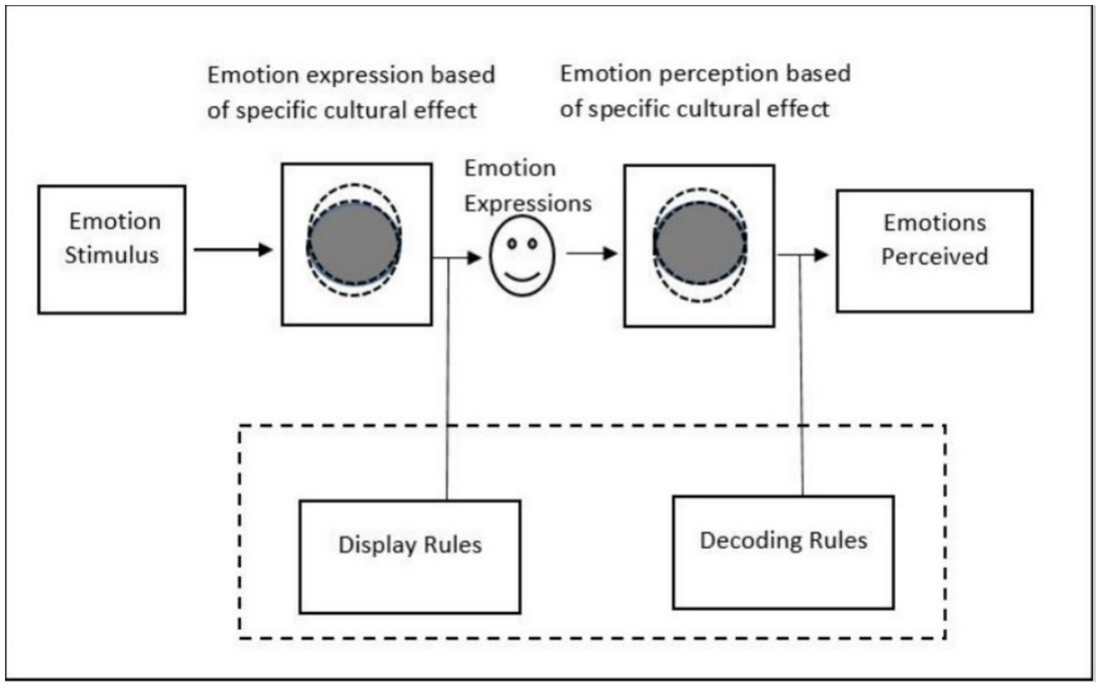
Dialect theory of emotions.

The metaphor of emotional dialect was first conceived by Tomkin in 1964 [13]. Just as language has different dialects which are variants of a language used by different speakers, separated by geographical distances, emotion also has dialects [14]. Emotional dialects mean slight variations in emotional displays with the change in culture [15] as depicted in Fig 1. The universality of emotions is represented by the gray circles and the effect of different cultures is represented by the partially overlapping white circles. The dashed box shows the sources of differences in the communication of emotions under the influence of some cultures. These variations are too subtle to be noticed, however, they may create misunderstandings for the people from another community. This is because like language dialects, emotional dialects also add flavor to the emotions which becomes difficult to perceive as accurately as an in-group member of that community. In dialect theory, there are two key points: i) The cultural differences must also be evident in emotion expression itself, and ii) Emotions expressed are more accurately perceived by the in-group members as compared to the out-group members. The rest of the paper is structured as follows. Section 2, reviews the related work with limitations. Section 3 presents materials and methods in terms of datasets and computational models. Section 4 shows our results for multi-lingual analysis and dialect theory of emotions in vocal expressions. Finally, Section 5 concludes this work.

## 2 Literature review

This section is devoted to the previous work on two perspectives: i) Psychological and ii) Technical. Psychological perspective highlights studies in which cultural differences in expression and perception of emotions using non-verbal cues, especially prosody are mentioned, whereas, technical perspective focuses on the application of machine learning for speech emotion perception to see cultural differences.

### 2.1 Psychological perspective

Researchers have explored different non-verbal cues, e.g facial features [16], eye movement [14], tone of voice [17], overall body movement [18], etc. for expression and perception of emotions. Specific to prosody, Teague studied prosodic emotion expression and perception, along with facial expressions, among three ethnic groups: Black Americans, Chinese Americans, and White Americans [19]. The results partially support the In-group advantage for facial expressions however, vocal expressions were not taken into consideration. Mandal studied emotion expression among different cultures using vocal cues [17]. Another study compared two distinct cultures English and Hindu for vocal emotion perception to see In-group advantage. The results supported the In-group advantage for the two cultures [20]. The scope of the study could be increased if more cultural groups were added. In a study [21] In-group advantage in emotion recognition through voice and face is studied between European Americans and African Americans. The results supported the In-group advantage more often by culture and less often by race. The studies [19, 20] which specifically focused on emotion perception, measured the response time of perceiver to analyze how quickly an emotional response is perceived. Moreover, the confidence level of the perceiver is also noted to achieve perception accuracy. The response time and confidence level of a person can be measured more precisely by using modern artificial intelligence means.

As the dialect theory of emotions has the liberty to integrate classic and recent findings [22], it is required to investigate emotional dialects by analyzing emotion data already available in the form of benchmark datasets. Moreover, emotion expression whether verbal or non-verbal is affected by cultural values [23]. As mentioned in [19], emotions other than face have been given very little attention, therefore, we are considering the audio emotion expression and perception to analyze emotional dialects among six cultural groups around the world using machine learning.

### 2.2 Technical perspective

For automatic speech emotion recognition, along with a suitable classifier, features are very important. Among two broad categories of speech features: linguistic and para-linguistic, the latter is more advantageous. The reason is, para-linguistic features can recognize speech emotions irrespective of which language is being used and what is being spoken [24]. Their only dependence is on the characteristics of speech signals such as tone, loudness, frequency, pitch, etc. In the study [1], to find emotion perception variation among different cultures, SVM-based classifiers were used. For this purpose, a large dataset was developed from 5 English-speaking cultures and In-group advantage was observed. This study was the first of its kind to use machine learning for observing cultural differences in vocal cues. However, this study was limited to using only English-speaking cultures, and also a large dataset was developed for this purpose which made this study quite expensive and time-consuming. To automate the process and to involve the diverse range of cultures, yet obtain the same results, there is a need to use already available datasets. A cross-lingual voice emotion recognition study was conducted using SVM based classifier [25]. The datasets considered in this study were multilingual. However, no findings concerning cultural differences in speech emotions were drawn. As speech features play important role in recognition, a speech-based emotion recognition task using nonlinear dynamic features in combination with prosodic and spectral was performed [26]. Berlin dataset was tested and a good gain of accuracy was achieved, however, the inclusion of more datasets may give more insight.

## 3 Materials and methods

Firstly, we will discuss the datasets of different cultures. Secondly, we will discuss the features and feature selection mechanism adopted in this research and thirdly, the machine learning model used for cross- cultural emotion perception will be discussed.

### 3.1 Datasets

We selected six datasets namely RAVDESS, EmoDB, ShEMO, SAVEE, EMOVO, and TESS of six different cultural areas as shown in Table 1. All the datasets are acted in style providing audio recordings annotated with the basic emotions. The number of actors varies in each dataset. For the sake of comparison, we took the equal number of recordings from each dataset and only those recordings which have common emotions in all datasets were considered. Also by considering only para-linguistic features, the impact of the verbal content of utterances was ruled out. Further detail of datasets, feature extraction, and feature reduction methods is as follows:

1. ***RAVDESS***: RAVDESS is an approved multi-modular database of emotional speech and song. There are 24 professional actors each uttering 104 unique intonations with emotions: happy, sad, angry, fear, surprise, disgust, calm, and neutral. The RAVDESS dataset is exceptionally rich in nature given that it doesn’t experience gender bias, comprises a wide range of emotions, and at various levels of emotional intensity. Each actor uses two different statements with two different emotional intensities, normal and strong for each emotion except for neutral which is with the normal intensity only. The total number of utterances is 1440.
2. ***EmoDB***: The EmoDB is an acted dataset of ten professionals (five male and five female) recorded by F. Burkhardt in the German language. It is labeled with seven emotion classes i.e. anger, boredom, fear, happy, disgust, neutral, and sadness. There are multiple utterances of the same speaker. Ten linguistically neutral sentences are chosen for dataset construction. Out of these 10 sentences, 5 sentences are short (approximately 1.5 Sec long) and 5 are long sentences (approximately 4 Sec long). Each emotion class has a nearly equal number of emotional utterances to avoid the problem of under-sampling emotion class. There are a total of 535 utterances in this dataset. It is one of the most widely used datasets in the literature [27]. The dataset includes those utterances which have a recognition rate of more than 80% in a subjective listening test.
3. ***ShEMO***: ShEMO is a large scale Persian language dataset [8] that contains 3000 semi-natural utterances. 87 native-Persian speakers were selected for emotional utterances of five basic emotions including anger, fear, happiness, sadness, surprise, and also neutral. The inter-annotator reliability was 64% and the validation of the dataset gave the best results with SVM for both gender-dependent and gender-independent models.
4. ***SAVEE***: SAVEE dataset provides audio utterances of British speakers [9]. The speakers were four British male actors who spoke the sentences showing six emotions: anger, sadness, disgust, happiness, surprise, fear. The sentences chosen were phonetically balanced. The dataset is comprised of 480 audio utterances. The data is processed and labeled under the visual media lab using high-quality audio and video equipment.
5. ***EMOVO***: It is an Italian language dataset based on an emotional corpus that consists of 6 actors. The number of utterances is 588 and 24 annotators of two different groups. To simulate seven emotional states, each actor has acted fourteen neutral short sentences.
6. ***TESS***: TESS is a Canadian English dataset that consist of 2800 utterances [11]. The speakers are two female actresses from Toronto Canada. The dataset is recorded at North Western University for an auditory test. Each recording consists of portraying seven emotions of happy, sad, anger, disgust, pleasant, fear, and neutral.

**Table 1:** Detail of datasets used for speech emotion recognition.

Database	RAVDESS [Ryerson 2018]	EmoDB [Burkhardt, 2005]	ShEMO [Nezami, 2019]	SAVEE [Jackson, 2014]	EMOVO [Costantini, 2014]	TESS [Pichora, 2020]
**No. of speakers**	24 (12 Males, 12 Females)	10 (5 Males, 5 Females)	87 (56 Males, 31 Females)	4 (Males)	6 (Males)	2 (Females)
**Language**	North American English	German	Persian	British English	Italian	Canadian English
**Style**	Acted	Acted	Acted	Acted	Acted	Acted

### 3.2 Preprocessing

In this step, datasets are preprocessed which involve audio reading, framing, and windowing. First, audio files are read, then unvoiced parts are filtered out, and ultimately, signals are framed in this part of preprocessing. After reading the signal, to eliminate the unvoiced and silent portion of the signal, Voice Activity Detection (VAD) algorithm is used. This step reads the audio file and converts it into frames, then checks VAD to each set of frames using Sliding Window Technique. The Frames having voices are collected in a separate list and non-voices (silences) are removed. Hence, all frames which contain voices are in the list are converted into preprocessed audio signal files. After that, it is required to opt for suitable feature extraction and selection mechanism.

### 3.3 Feature extraction

Once preprocessing is done, we moved towards feature analysis which involves the selection and extraction of useful features for speech emotion analysis. Here we are only considering para-linguistic features. Well known toolboxes for feature extraction in speech signals are OpenSMILE [28], OpenEAR [29], HTK [30], and Praat [31]. In this research, OpenSMILE toolbox is used to extract INTERSPEECH 2010 Challenge feature set [32]. The reason for opting INTERSPEECH 2010 feature set is because, it covers most of the features namely (prosodic, spectral, and energy) effective for emotion recognition. This fact was proved in [33] where it gave the best results for emotion recognition with many classifiers. The technical description of the important features is given as below.

Prosodic features include energy and zero-crossing rate. The short term energy is computed for each frame as:

$$\begin{array}{c} E_{n}=\Sigma_{i=-\infty }^{\infty }\left(x_{\left(i\right)}\right)w\left(n-i\right)) \end{array}$$ (1)

One of the frequently used Spectral features is formants frequencies. Multiple filters are applied to voice signals as they travel down the vocal cord according to Fant’s model. Mathematically, filter equation may be formulated as:

$$\begin{array}{c} E(Z)=U(Z).V(Z).K(Z) \end{array}$$ (2)

where is V is vocal tract filter, U is glottal plus scaled by voice controller and K is lip radiation filter. The voice quality features such as jitter and shimmer are calculated by fundamental frequency and cycle to cycle variation in voice intensity. The mathematical formula for measuring jitter is as follows:

$$\begin{array}{c} jitter_{r}\left(i\right)=\frac{\frac{1}{N-1}\sum_{i=1}^{N-1}\left|T\left(i+1\right)-T\left(i\right)\right|}{\frac{1}{N}\sum_{i=1}^{N}\left|T\left(i\right)\right|} \end{array}$$ (3)

$$\begin{array}{c} jitter_{r}\left(i\right)=\frac{\frac{1}{N-1}\sum_{i=1}^{N-1}\left|T\left(i+1\right)-T\left(i\right)\right|}{\frac{1}{N}\sum_{i=1}^{N}\left|T\left(i\right)\right|} \end{array}$$ (4)

Where T(i) is the wavelength of the fundamental frequency F0, where N is the number of extracted periods of F0. The shimmer satisfy the following mathematical form.

$$\begin{array}{c} shimmer_{r}\left(i\right)=\frac{\frac{1}{N-1}\sum_{i=1}^{N-1}\left|A\left(i+1\right)-A\left(i\right)\right|}{\frac{1}{N}\sum_{i=1}^{N}\left|A\left(i\right)\right|} \end{array}$$ (5)

Where A(i) is the extended peak to peak amplitude data and N is the number of extracted frequency period.

### 3.4 Feature optimization and reduction

As the feature set is huge, consisting of 1583 features, we need a good dimensionality reduction technique. Before reducing the size of features, we will optimize features so that good prediction results could be achieved. Genetic algorithms have great benefit for applying at feature selection stage [34]. Their two powerful functions: cross-over and mutation have the ability to optimize the features which will increase the perception accuracy.

Among the effective methods available for feature reduction, Forward Feature Selection (FFS), Backward Feature Selection (BFS) [35], Principle Component Analysis (PCA), and Linear Discriminate Analysis (LDA) [36, 37], we opted for the most commonly used PCA [38–40]. PCA includes finding the eigenvalues and eigenvectors of the available covariance matrix, and choosing the necessary number of eigenvectors compared to the biggest eigenvalues to create a transformed matrix. The matrix is utilized to change the original feature set into a transformed feature space and select the best-required features. We applied the genetic algorithm to the feature set (INTERSPEECH 2010 feature set) and get the optimized set having the same dimensions. After that, the feature set is fed to PCA to get the reduced feature set consisting of 100 features that have further been used in the classification step.

### 3.5 Machine learning model

**Fig 2:**
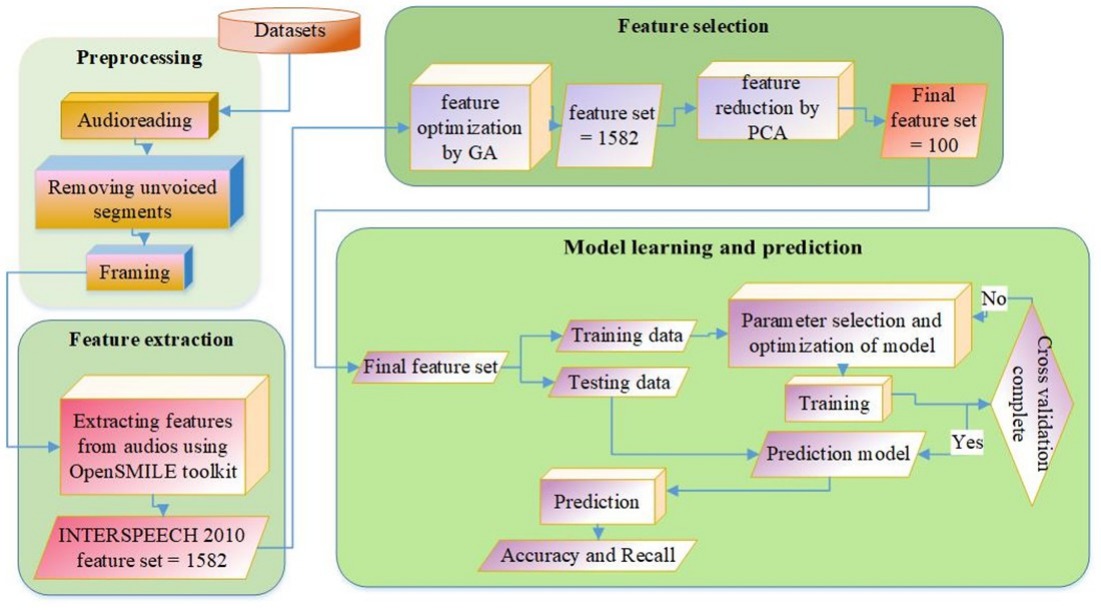
Computational model.

To study multicultural emotion perception and In-group advantage through machine learning, we have divided the process into two phases: Inter-culture emotion perception and Intra-culture emotion perception. In inter-culture emotion perception, the SVM model trained using one cultural group is tested with the rest of the cultural groups one by one. Applying all classifiers to an unseen sample x and predicting the label k for which the corresponding classifier reports the highest confidence score: In this way, the emotion perception accuracy is noted. This process is repeated for all cultural groups. In intra-culture emotion perception, as samples are from the same cultural group, we have adopted the Leave-one-subject-out strategy. By doing so, the model is trained using n-1 subjects and tested with the nth subject of the same cultural group. By doing so it is hoped that In-group advantage could be seen accurately. The complete model from preprocessing to classification is represented in Fig 2.

## 4 Experiments and results

In this section, multicultural emotion perception and in-group advantage will be discussed.

### 4.1 Emotion perception of British speakers

**Fig 3:**
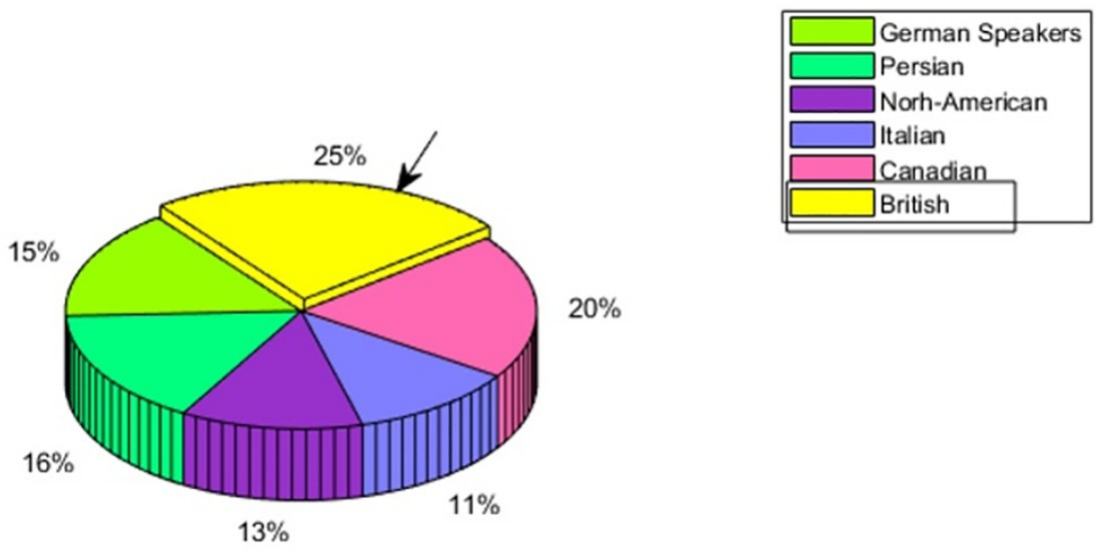
British speaker—Multicultural emotion accuracy comparison and In-group advantage.

For the general emotion perception comparison of British speakers, the model is trained with British speakers’ audio features. This trained model is tested with the audio features of Persian, North-American, Italian, Canadian and German speakers. For analyzing the In-group advantage in the case of British speakers, one speaker is taken out to train the model and an unknown speaker was exposed to the training data of the British culture. the In-group advantage was observed in the case of British speakers having the recognition rate of 25% as compared to 20% for Canadian speakers, 16% for Persian speakers, 15% for German speakers, 13% for North-American speakers, and 11% for Italian speakers. Based on this observation, it can be concluded that the In-group advantage was there for British speakers. This is represented in Fig 3.

**Fig 4:**
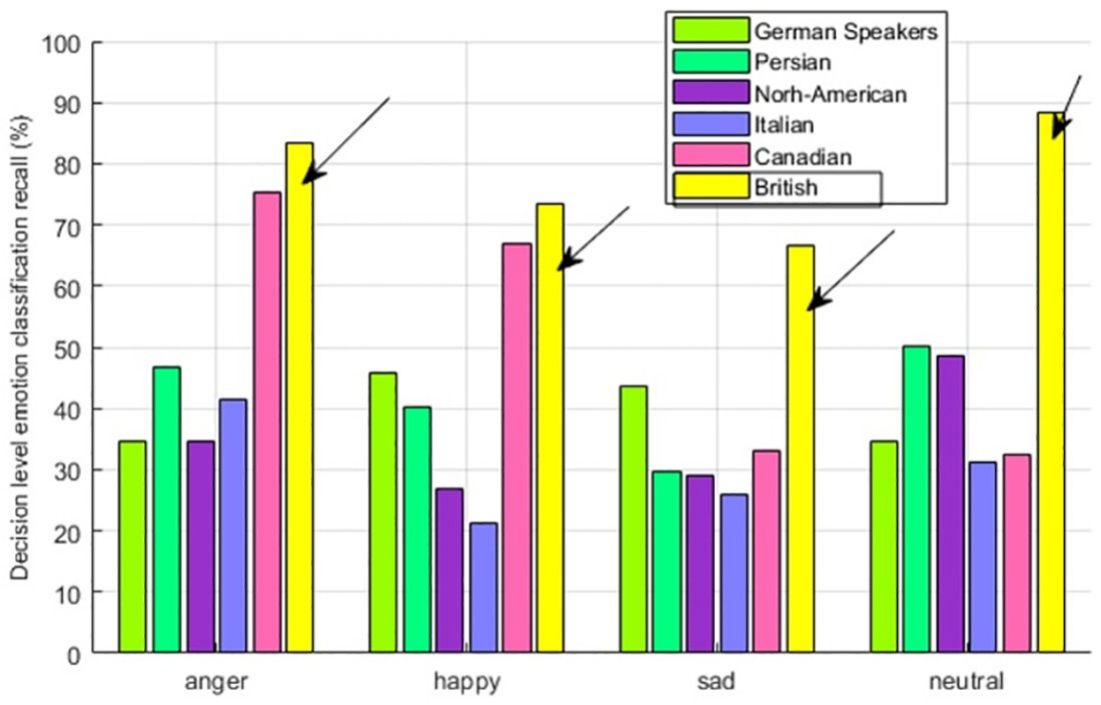
British speaker—Multicultural emotion recall comparison and In-group advantage.

In order to see the emotion perception accuracy of multiple cultures for individual emotions, we have calculated the emotion-wise recall. As shown in Fig 4, four emotions of anger, happiness, sadness, and neutral are showing the highest emotion recall for British speakers. This observation indicates the evidence of In-group advantage and also that British speakers are more inclined to their own cultural dialects and that people of British culture are more accurate at perceiving the emotions of their own cultural community.

#### 4.2 Emotion perception of Canadian speakers

**Fig 5:**
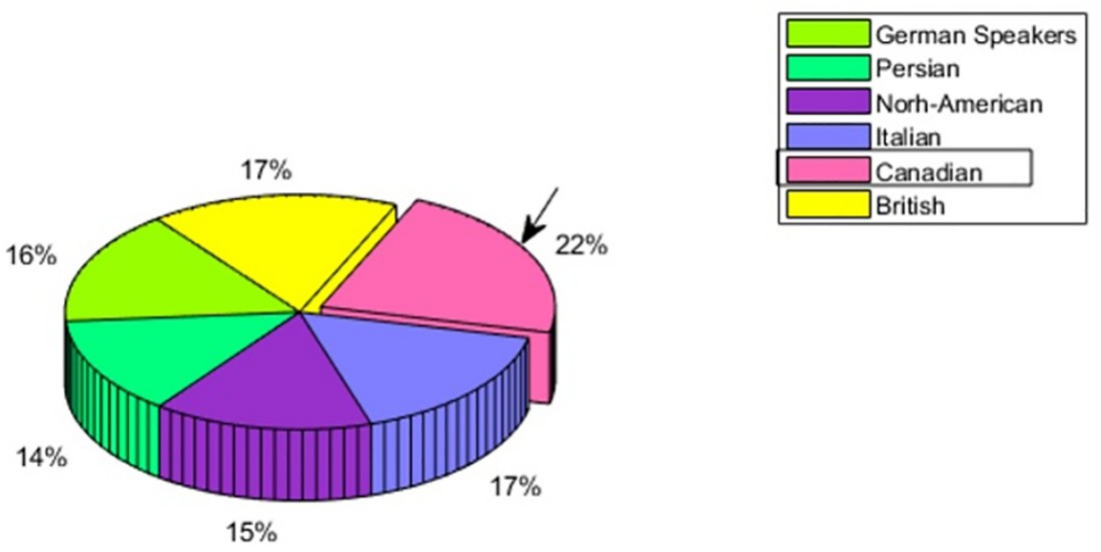
Canadian speaker—Multicultural emotion accuracy comparison and In-group advantage.

For the Canadian culture, Fig 5 depicts the multicultural emotion perception comparison.

**Fig 6:**
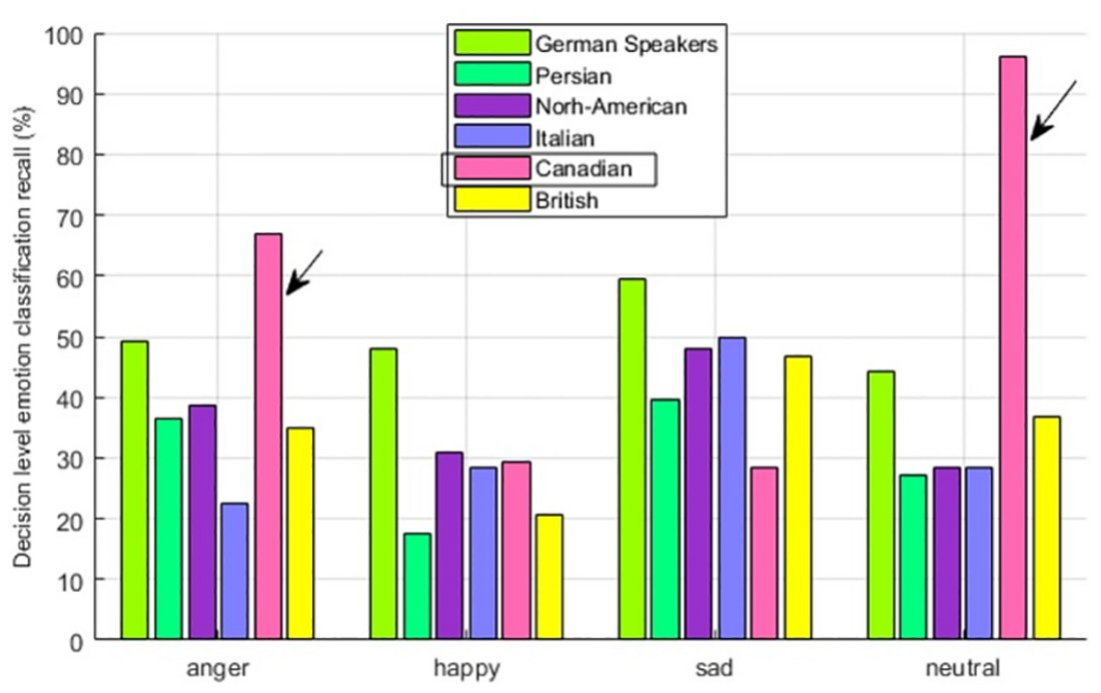
Canadian speaker—Multicultural emotion recall comparison and In-group advantage.

Here the model is trained with the audio features of Canadian speakers’ audio utterances and tested with German, Persian, North-American, Italian, and British speakers’ utterances. The general emotion perception results demonstrate the evidence of In-group advantage having the emotion perception rate of 22% for Canadian speakers. As compared to this, the North-American and British have a recognition rate of 17%, German speakers having 16%, and Persian speakers having 14%. The North-America being located near to Canada is showing the second nearest perception accuracy which is also giving the evidence of In-group advantage. The emotion wise perception recall is seen in Fig 6. Out of four emotions, the In-group advantage is evident for anger and neutral, whereas, for emotions happiness and sadness, German speakers are showing the highest accuracy. So for Canadian speakers, emotion wise In-group advantage is partially seen.

#### 4.3 Emotion perception of German speakers

**Fig 7:**
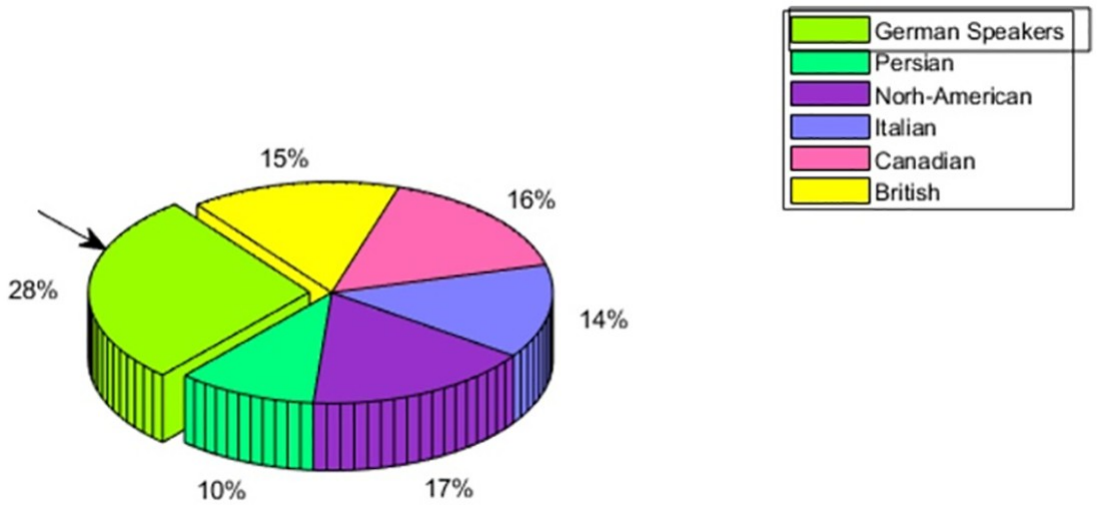
German speaker—Multicultural emotion accuracy comparison and In-group advantage.

**Fig 8:**
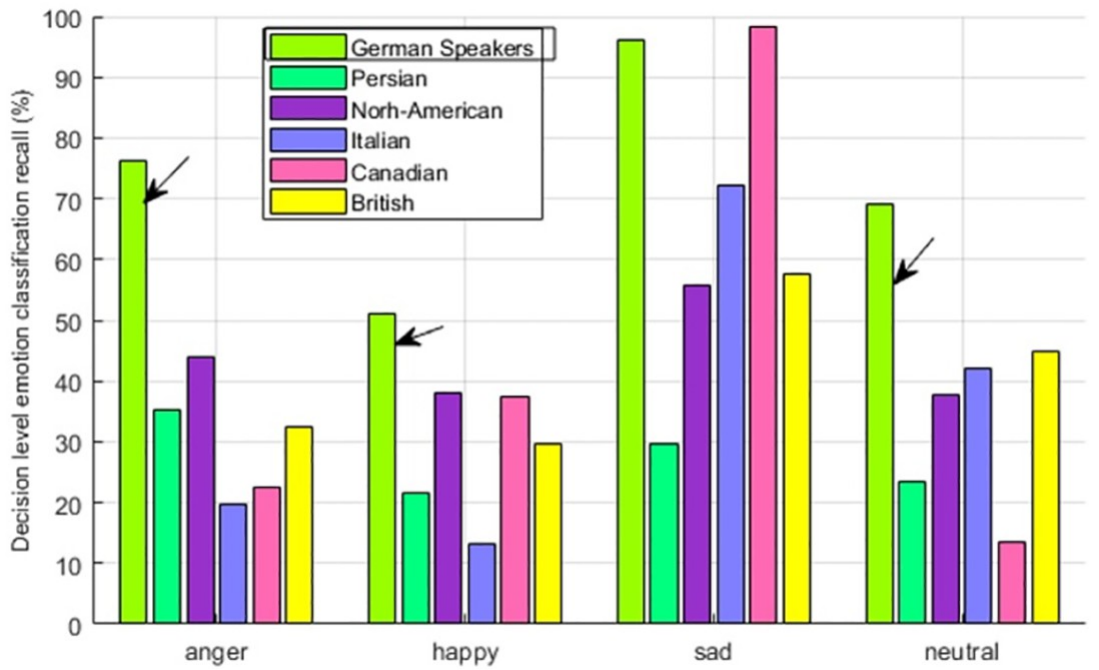
German Speaker—Multicultural Emotion Recall Comparison and In-group Advantage.

German speakers are showing the obvious in-group advantage of 28% as compared to North-American having 17%, Canadian having 16%, British having 15%, Italian having 14%, and 10% for Persian. This is depicted in Fig 7 Upon the detailed view of emotion perception, it is seen from Fig 8 that, for three emotions, anger, happiness, and neutral, it is showing the highest perception accuracy for its own people. For the emotion sadness although the perception accuracy for In-group people is high i.e. above 90%, however, Canadian speakers are showing the highest perception accuracy of the said emotion.

#### 4.4 Emotion perception of North-American speakers

**Fig 9:**
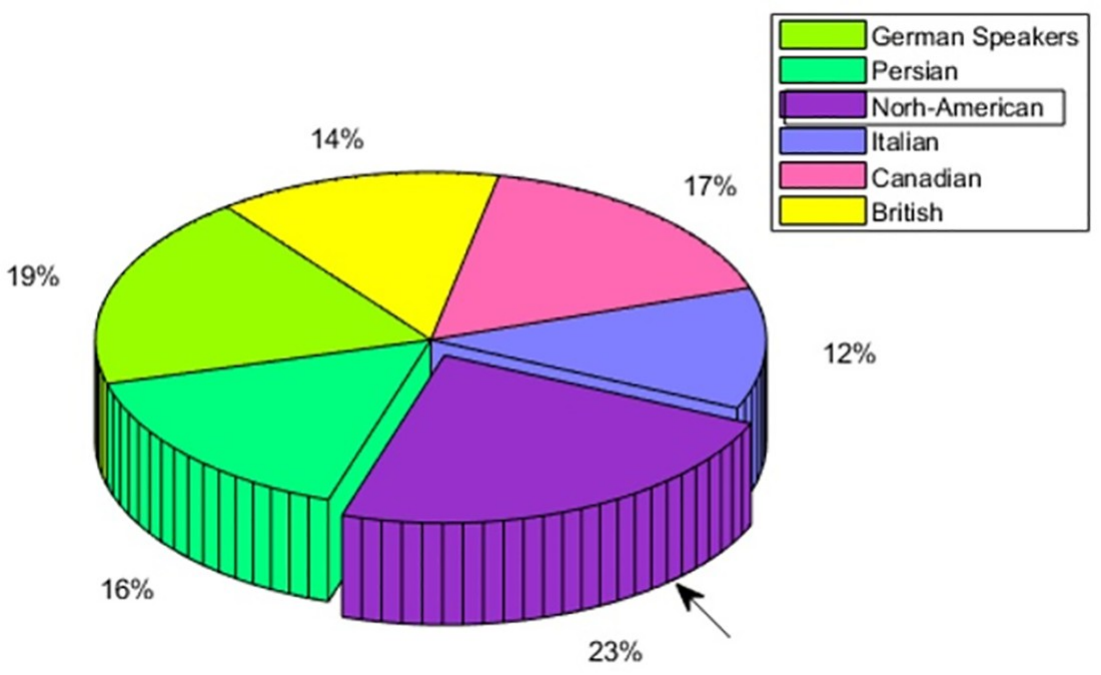
North-American speaker—Multicultural emotion accuracy comparison and In-group advantage.

The people of North America are also showing the In-group advantage having an overall perception accuracy of 23% as compared to 19% for German culture, 17% for Canadian culture, 16% for Persian culture, 14% for British culture, and 12% for Italian culture. This is shown in Fig 9.

**Fig 10:**
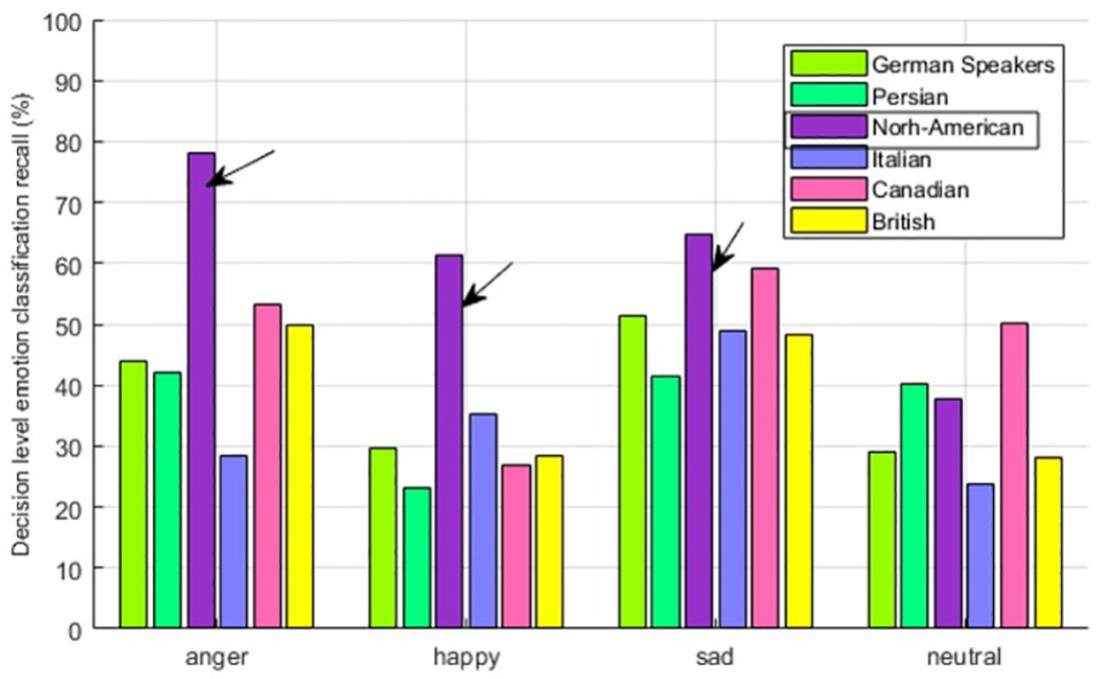
North-American speaker—Multicultural emotion recall comparison and In-group advantage.

The emotion-wise recall comparison for North American people in Fig 10 highlights the In-group advantage of anger, happiness, and sadness. For neutral, it is not showing the In-group advantage.

#### 4.5 Emotion perception of Persian speakers

**Fig 11:**
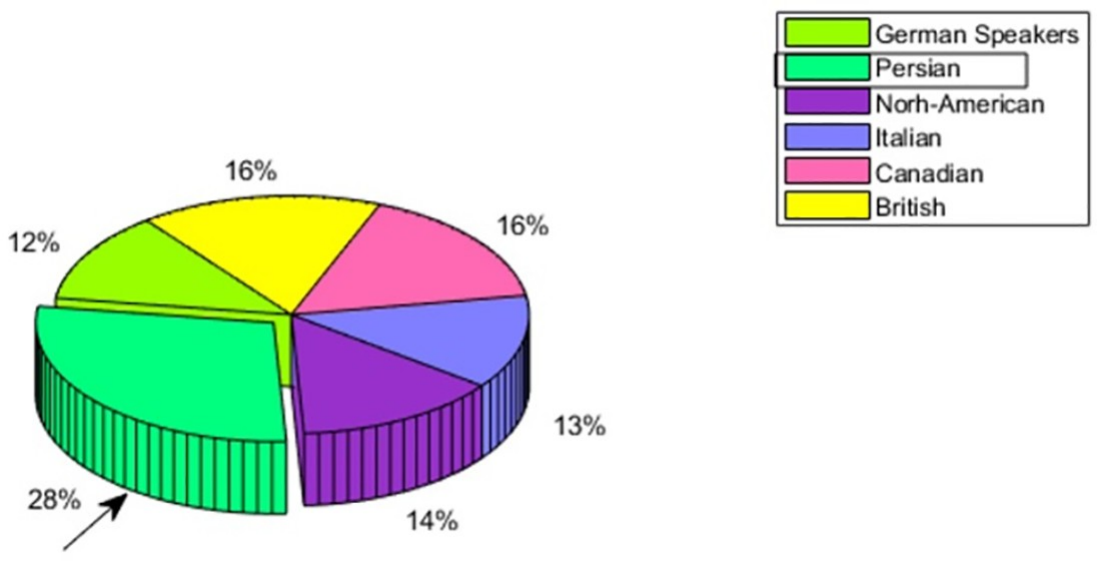
Persian speaker—Multicultural emotion accuracy comparison and In-group advantage.

**Fig 12:**
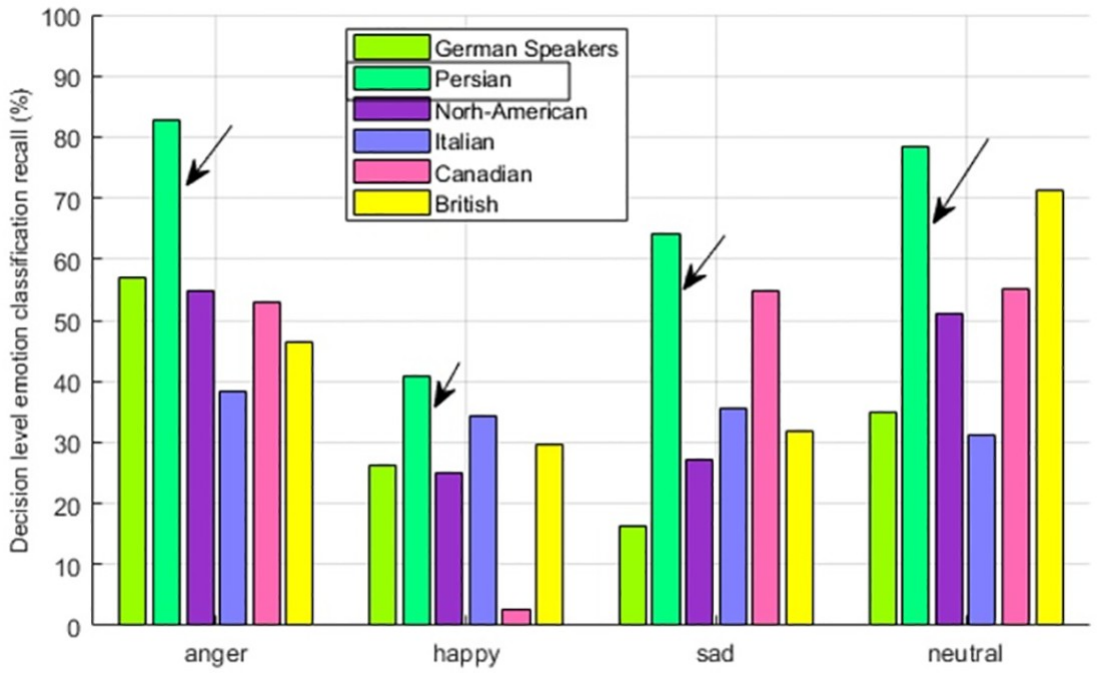
Persian speaker—Multicultural emotion recall comparison and In-group advantage.

The emotion perception accuracy of Persian speakers is shown in Fig 11. The native speakers are showing the above-average emotion perception accuracy of 28% which is fairly higher than the rest of the cultural groups. If we analyze the emotion perception of individual emotion given in Fig 12, all are showing the distinct In-group advantage. This fact leads to the conclusion, that Persian speakers adhered to their own culture more than any other.

#### 4.6 Emotion perception of Italian speakers

**Fig 13:**
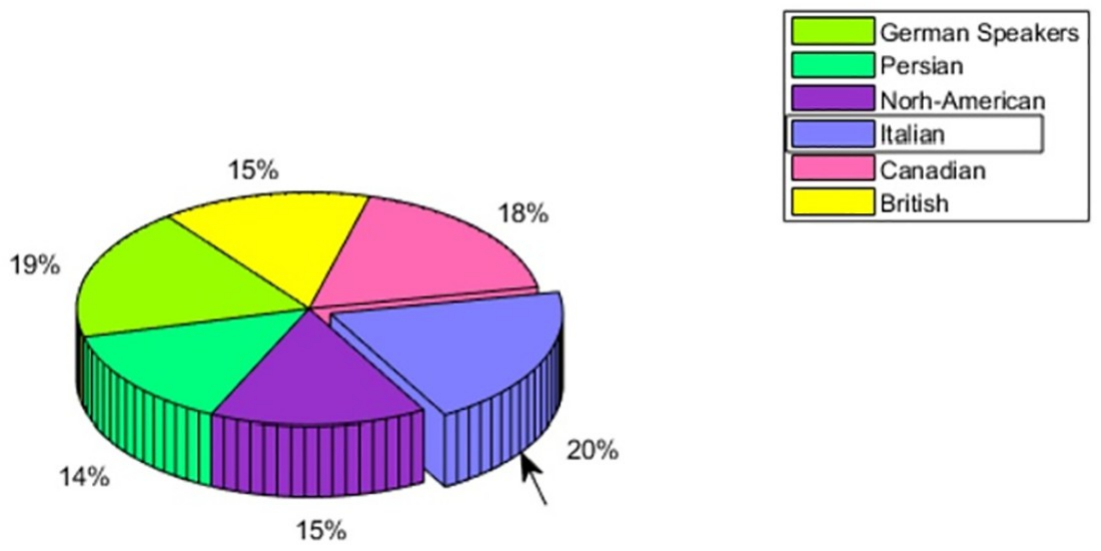
Italian speaker—Multicultural emotion accuracy comparison and In-group advantage.

The Italian speakers are showing the In-group advantage of 20%, however, the second nearest to it is of German speakers which are of 19% depicted in Fig 13.

**Fig 14:**
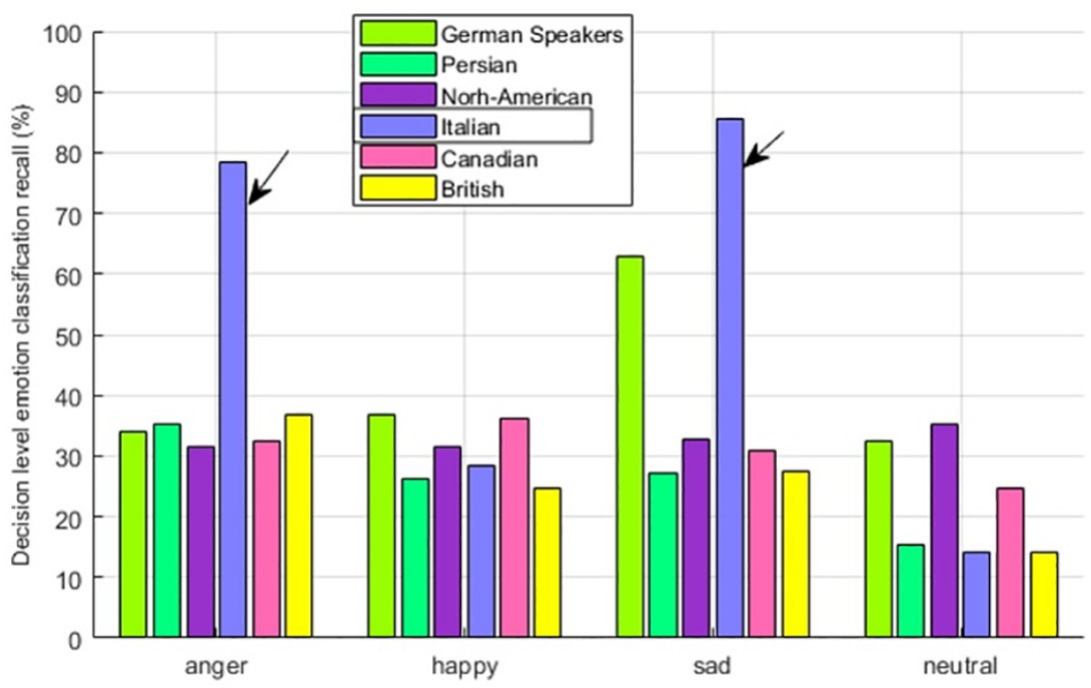
Italian speaker—Multicultural emotion recall comparison and In-group advantage.

The one reason could be that geographically, the two cultural groups are living near each other. Detailed analysis of emotions show that anger and sad emotions are having remarkable In-group advantage, however, happy and neutral are hardly showing any evidence of In-group advantage as seen from Fig 14.

## 5 Conclusion

Results drawn from the experiments showed that overall classification accuracy for emotion perception was consistently higher for with-in group people. It means when training and testing sets were taken from the same culture, we have received a high emotion perception accuracy for different cultures. The emotion-wise classification recall of cultures gave evidence that partially proved the In-group advantage. This is because regardless of culture, emotions also have universal recognition [41, 42]. However, most of the emotions showed an In-group advantage confirming the dialect theory of emotions. Cross-cultural audio emotion comparison results provide a systematic explanation of how people perceive different emotions due to the natural culture gap rather than any prejudice or difference. To increase the familiarity of unknown cultures, training programs can be arranged. In this way, In-group advantage can be eliminated.
